# Tackling barriers to collective action for effective vaccination campaigns: rabies in rural Africa as an example

**DOI:** 10.1057/s41599-022-01374-3

**Published:** 2022-10-12

**Authors:** Putthi Cheat Lim, Tiziana Lembo, Katie Hampson, Joel Changalucha, Maganga Sambo, Sayantan Ghosal

**Affiliations:** 1 Adam Smith Business School, University of Glasgow, Glasgow, UK; 2 The Boyd Orr Centre for Population and Ecosystem Health, Institute of Biodiversity, Animal Health & Comparative Medicine, College of Medical, Veterinary & Life Sciences, University of Glasgow, Glasgow, UK; 3 Environmental Health and Ecological Sciences Department, Ifakara Health Institute, Ifakara, Tanzania

**Keywords:** Health humanities, Economics

## Abstract

Vaccine-based protection in populations that are vulnerable to infectious diseases represents a public good, whose successful attainment requires collective action. We investigated participation in mass domestic dog vaccination against dog-mediated human rabies endemic in Tanzania as a prototypical example of these issues. We employed advertising interventions, text messaging and/or engagement through community leaders, as well as operational adjustments to increase the saliency of rabies risks and reduce barriers to participation in vaccination campaigns. Neither advertising strategies were effective on their own, however, when taken together, the two advertising strategies substantially improved vaccination coverage. Operational interventions, such as increasing vaccination stations and extending time windows of delivery, greatly enhanced participation. Our experimental and theoretical findings highlight the importance of both salience and context: sparking successful collective action requires decision-making bodies to understand and respond to the challenges encountered by intended beneficiaries in their local contexts.

## Introduction

Collective participation is a prerequisite for generating public goods that benefit communities as a whole (Olson, 1965). The provision of such public goods is critical in many contexts, from clean water and food reserves provided by intact forests, mangroves and fisheries, to improving a population’s health and livelihoods and in disease prevention (Bardhan, 2000; Gautam, 2006; Jentoft and Finstad, 2018; Siegal et al., 2009). Even when a government agency initially funds a public good, collective action is essential in order for it to generate benefits for a community over time. For instance, community members need to work together to maintain irrigation systems, build dispensaries and keep their villages clean.

In the context of public health, an important public good, particularly for under-resourced and marginalised communities, is the elimination of infectious diseases that cause preventable mortality, morbidity and production losses. Evidently, vaccination provides individual-level protection but eliminating a disease to protect an entire community requires achieving a threshold level of vaccination coverage. When the proportion of the immunised population exceeds this critical threshold, herd immunity is attained, which prevents the occurrence of large outbreaks and leads to elimination (Fine, 1993). Hence, disease elimination requires community-wide collective action. Here, we examine the control of rabies through dog vaccination as a prototypical example of community-wide collective action.

Rabies causes approximately 59,000 human deaths annually across the world (Hampson et al., 2015), with the greatest burden falling in Africa and Asia, mostly due to bites from infected domestic dogs. The populations most affected by rabies are typically marginalised rural communities, mostly children, who have limited access to health care and are least able to pay the costs required for prevention (Sambo et al., 2013). Human deaths from rabies can be effectively prevented through timely administration of post-exposure prophylaxis after someone is bitten (World Health Organization, 2013). However, post-exposure prophylaxis is often not accessible and expensive (Changalucha et al., 2019). In contrast, mass dog vaccination against rabies is cost-effective and addresses the problem at source, with broader and long-term benefits derived from the elimination of disease (Lankester et al., 2014).

Successful dog vaccination campaigns have been shown to reduce bites by rabid dogs and demand for post-exposure prophylaxis (Cleaveland et al., 2003). Moreover, rabies can be eliminated if at least 70% of the dog population is vaccinated (Coleman and Dye, 1996). Once elimination is achieved, the source of infection to humans is also removed and hence the risk of rabies.

Sustained dog mass vaccination campaigns have eliminated rabies from many parts of the world (Vigilato, 2013). However, the required vaccination coverage has not always been achieved, even in communities most at risk. Reasons for this failure may pertain to the demand side, i.e., sub-optimal participation in vaccination campaigns by community members, or the supply side, i.e., issues around the availability and delivery of vaccines, or both.

Many factors can influence the demand for canine rabies vaccines and hence participation in vaccination campaigns. For instance, living in poverty may impact on the salience of rabies as a potential health problem due to the multitude of other challenges in people’s everyday lives. Perceptions around the importance of rabies compared to other health issues or of immunisation programmes targeting dogs rather than more economically valuable species such as livestock may also alter willingness to participate. It is critical to consider how rabies interventions fit amongst competing priorities confronting those whose participation is required to achieve the desired coverage, and how to encourage participation. Other demand-related factors include, for example, the cost of participation, awareness about rabies, knowledge of vaccination campaigns and the capability of each community to solve the collective action problem. On the supply side, infrastructural challenges affecting vaccine availability and accessibility are likely to affect participation. Operational factors have indeed been shown to be barriers in the context of dog vaccination (Bardosh et al., 2014; Beyenne et al., 2018; Castillo-Neyra et al., 2019).

The research reported here focuses on the demand for canine rabies vaccines. It describes the results of two interventions using two different communication strategies for ensuring the salience of, and participation in, dog vaccination campaigns within a community: mobile phone text messaging and promotion by community leaders. In addition, operational changes to ease access to vaccination campaigns for villagers were introduced. The combination of these interventions was designed to overcome the collective action problem by increasing the salience of rabies as a health problem, and by reducing the cost of participation in vaccination campaigns. Our theoretical framework (see Box 1) models the effect of different interventions on the solution to the underlying community-wide collective action problem, which determines the level of participation in a vaccination campaign.

Box 1 Theoretical framework.

Consider an environment with *N* agents, where *N* is finite but very large (so that a version of the law of large numbers applies). Let *γ*, where 0 ≤ *γ* ≤ 1, represent the proportion of the agents informed about the vaccination campaign. Hence, *M* = *γN* is the number of agents informed about the vaccination campaign, where it is assumed that *M* is a whole number. Informed agents must make up to two decisions. First, they must decide whether to pay attention to the vaccination campaign. Second, if they decided to pay attention to the campaigns, they must decide whether to participate in the campaigns. The pay-off for their decisions are as follows:

*Decision 1:*

- If agent *i* does not pay attention to the campaigns, then he simply gets a payoff of $k_i \in \left\{ {\underline k ,\overline k } \right\}$, where $\underline k < \overline k$. Here, *k*_*i*_ is interpreted as the payoff from paying attention to other activities and captures the opportunity cost of paying attention to the vaccination campaign. We assume that a fraction *s* of the informed agents draw $k_i = \underline k$ while a fraction 1−*s* draw $k_i = \overline k$.
  If agent *i* decides to pay attention to the campaigns, then he must make the second decision, which is whether to participate in the campaigns:
*Decision 2:*
- If agent *i* participates, then he gets a payoff of $p\left( {L^\prime } \right) + \alpha - c_i$, where *p*(*L*′) is the probability of the public good being produced given *L*′ (the total number of participating agents), *α* is the private benefit of participation and *c*_*i*_ is the cost of participation. It is assumed that $c_i{\it{\epsilon }}\left\{ {\underline c ,\overline c } \right\}$, where $\underline c < \alpha < \overline c$. We also assume that of those who participate, a fraction *t* draws $c_i = \overline c$ while a fraction 1−*t* draws $c_i = \overline c$.
- If agent *i* does not participate, then he simply gets a payoff of *p*(*L*′).

***Solution:***

The model is solved by backward indication. At *Decision 2*, under the assumptions made, a fraction *t* of those who chose to pay attention at *Decision 1* would choose to participate. At *Decision* 1, note that an agent who chooses to pay attention will also decide to participate at *Decision 2*, because there is an opportunity cost to paying attention, and incurring the opportunity without choosing to participate is dominated by not paying attention to the vaccination in the first place. Hence, the payoff of paying attention can be written as $p\left( {tM} \right) + \alpha - \underline c$ because only those agents with $c_i = \underline c$ will be willing to participate. The payoff of not paying attention is simply *k*_*i*_. At this stage, an agent’s decision then depends on his value of *k*_*i*_. An agent chooses to pay attention and participate in the campaigns if $p\left( {tM} \right) + \alpha - \underline c \ge k_i$ and not, otherwise. s^*^ that denotes the fraction of agents who pay attention to the vaccination campaign is determined as follows:

- $$s^ \ast = 1\,{\mathrm{if}}\,\underline k\, <\, \overline k\, < \,p\left( {tM} \right) + \alpha - \underline c$$
- $$s^ \ast = s\,{\mathrm{if}}\,\underline k\, <\, p\left( {tM} \right) + \alpha - \underline c \,<\, \overline k$$
- $$s^ \ast = 0\,{\mathrm{if}}\,p\left( {tM} \right) + \alpha - \underline c \,<\, \underline k \,<\, \overline k$$

Hence, the number of participants in the vaccination campaign will be γs*tN.

The model predicts that participation in the vaccination campaign can be increased by changing the values of three parameters. First, an intervention can increase the value of *γ*, which is the number of informed participants. An example would be using novel communication interventions as in our experiment below. Second, an intervention can lower the opportunity cost of paying attention to the vaccination campaign and/or lower the cost of participating in the vaccination campaign (and hence, the values of *s*, *t* and s^*^). An example of such an intervention is the operational changes in our experiment.

## Results

### Interventions altering the salience of canine vaccination

An intervention to raise participation of dog-owners in canine vaccination campaigns to eliminate rabies, and therefore achieve the public good, needs to do more than just inform potential participants about its benefit. It needs to make the risk of rabies salient to the intended audience and reduce participation costs. For achieving salience, we used two advertising methods, namely text messaging and engagement by community leaders. Community leaders here refer to religious leaders from local Muslim and Christian communities and local Maasai leaders. We hypothesised that both advertising interventions have potential to provide relevant information about a vaccination campaign and make it salient. Advertising through text messaging and/or community leaders may have greater reach than routine advertising methods for vaccination campaigns, such as posters or loudspeakers. Moreover, community leaders may leverage their authority, to act as a focal point for the solution to collective action problems within their communities, as demonstrated in other situations (Vedeld, 2000; Glowacki and von Rueden, 2015).

The design of the interventions was as follows. For the text messaging intervention, phone numbers were collected manually. A team of officials were assigned villages to record phone numbers of as many households as they could, which were then imported into a mass text messaging platform (Rasello). A series of workshops and focus group discussions (see Methodology) were used to design the content and delivery timing of the following text message:

“**VILLAGE NAME:** Recently, in a neighbouring village to yours, a child died a painful death after being bitten by a rabid dog. You and your neighbours can protect your children by vaccinating your dogs. Dog vaccines in **VILLAGE NAME** will be provided for free at **LOCATION(s)** on **DAY DATE** between 8 am to 4 pm.”

In the message the village name was capitalised to emphasise to text recipients that the message related to an issue relevant to their village. The first sentence was an anecdote of a real event that was meant to make the risk of rabies salient to the recipients. The next sentence encouraged recipients to inform and encourage others to vaccinate their dogs. The final sentence informed the recipients of the location and timing of the vaccination campaigns and that the vaccines were provided for free. The text-messages were sent 5 days, 3 days and 1 day before the campaigns to give recipients time to prepare and to remind them of the campaigns. The text-messages were sent at approximately 4 pm local time as this was discussed to be a suitable time when villagers usually finish their daily work and interact socially.

Before the start of the campaigns, religious and Maasai leaders in the assigned villages were identified by the district veterinary officers for the community leader intervention. These leaders were sent a letter at least 1 week before the vaccination campaigns inviting them to advertise the campaigns to their community members. The letter included a sample message, which they could use:

“To eliminate rabies from our village and to protect our family and friends from rabies, it is important that we all bring our dogs to be vaccinated in the upcoming vaccination campaign. If all dogs in our village are protected against rabies, then we will not be at risk of contracting the deadly disease. Vaccinating your dogs against rabies is the right thing to do. The next rabies vaccination campaign will take place in our village on **DAY DATE** at **LOCATION(s)** between 8 am to 4 pm.”

It should be noted that the above message was only a sample to guide the community leaders. The community leaders were made aware that they had the freedom to advertise in any way they wanted (the timing, the frequency and the content of their message), and that they should clearly communicate the timing of the campaigns and that vaccination would be provided free of charge. These leaders were sent reminder text-messages throughout the week, including before their weekly meetings. Our data indicated that community leaders in all assigned villages advertised as requested.

To maximise consistency in the effect of the advertising interventions across all 56 sampled villages, we made three operational changes to how vaccination campaigns were run. First, we ensured that vaccination points were placed in each sub-village. This tended to equalise the average distance villagers had to walk and the time for them to reach vaccination points and therefore the opportunity cost of the time and effort required to participate in the vaccination campaign across villages. Second, vaccination points were open all day long. Previously, vaccination points were usually open for only half a day. This meant that in our campaigns there was no bias between villages with points open in the morning or in the afternoon. Thirdly, routine advertising, such as posters and loudspeakers, began 1 week before the vaccination campaigns, to provide ample time for information to spread throughout the villages. These changes increased comparability between our sampled villages but also reduced participation costs directly.

**Fig. 1:**
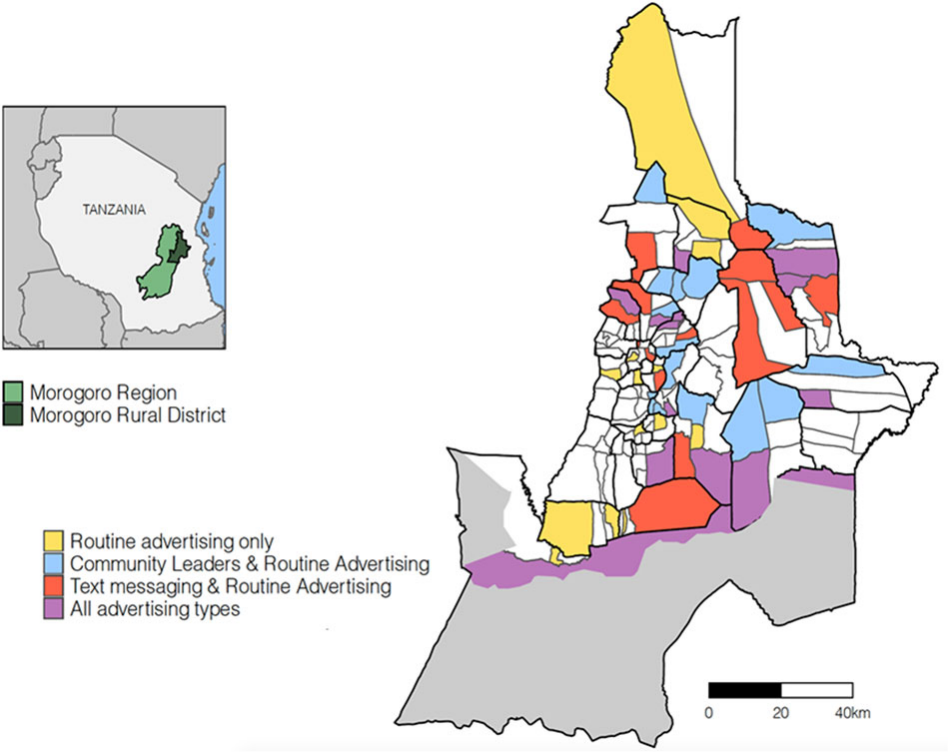
Villages in the Morogoro Rural District in eastern Tanzania. All coloured villages were part of our experiment and received different advertising interventions according to the legend. White villages were not part of our study and did not receive any of our study interventions. However, vaccination campaigns in those villages were held 1 day after the villages in our study. Protected areas, which are uninhabited, are shown in grey.

We tested these advertising interventions in 56 villages in the Morogoro Rural district of Tanzania. These villages, comprising 43% of the district’s population with 122,945 inhabitants (National Bureau of Statistics and Office of Chief Government Statistician, 2013), were randomly selected from 93 villages in the district with confirmed mobile phone network. Each village was randomly assigned to one of four groups (Fig. 1): routine advertising only (*Routine Advertising Only*), routine advertising plus either advertising by community leaders (*Community Leaders Only*) or text messaging (*Text Messaging Only*) or by both text messaging and community leaders (*All Advertising Types*). District authorities held dog vaccination campaigns in all 132 villages in the district, including the 56 study villages, from January 20th to January 21st of 2018, with campaigns in the sampled villages on the 20th January. A total of 7210 dogs were vaccinated, with 3256 vaccinated from sampled villages. We estimated vaccination coverage achieved in these villages using post-vaccination transects (Sambo et al., 2017).

**Fig. 2:**
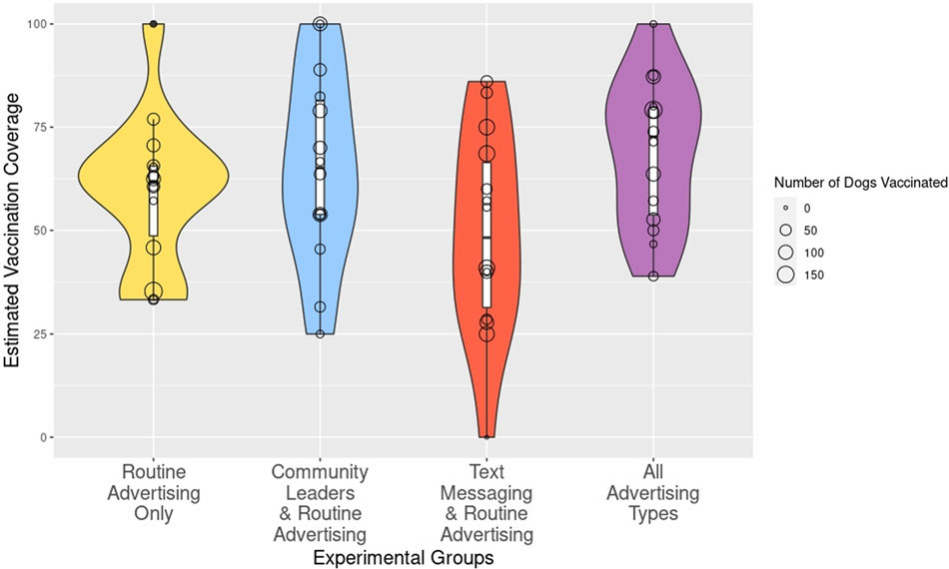
Mean vaccination coverage estimated from post-vaccination transects, variation for all four experimental groups and numbers of dogs vaccinated in each study village. Villages that received both interventions had the highest mean vaccination coverage, at ~70%, whereas the group receiving text messaging had the lowest at around 50%. Average vaccination coverage in the group with only routine advertising and the group with routine advertising plus community leaders was 60% and 65%, respectively.

We found no evidence that either of the two advertising interventions, on their own, had a statistically significant (at 10% level) impact on participation in vaccination campaigns, but when used together we found they could increase participation (Fig. 2 and Table 1, detailed discussion below). First, by looking at Fig. 2, the mean vaccination coverage in the villages receiving *Routine Advertising Only*, *Community Leaders Only* and *Text Messaging Only* were not significantly different, especially given the large within-group variation. However, the mean vaccination coverage in the villages that received both advertising interventions was higher than in the other villages with significantly less variation.

**Table 1:** Generalised linear mixed models examining the effect of the advertising interventions on participation in vaccination campaigns, with estimated vaccination coverage from transects transformed using the logit link function as our dependent variable.

Independent variables	Model 1 coefficient (standard error)	Model 2	Model 3
Text messaging coverage	−0.1023 (0.4162)	−1.0439^a^ (0.6116)	−1.0391^a^ (0.6067)
Community leaders	0.4601^b^ (0.2329)	0.0429 (0.3011)	0.0518 (0.2986)
Pastoralist presence	−0.2594 (0.2454)	−0.3191 (0.2404)	−0.2778 (0.2421)
Livestock field officer presence	0.4803 (0.3995)	0.6931^a^ (0.4020)	0.7376^a^ (0.4015)
Collective action indicator			−0.6769 (0.7152)
Text messaging Coverage*Community Leaders		1.7399^b^ (0.8405)	1.6772^b^ (0.8353)
AIC	283.9	281.7	282.8

Numbers in brackets are the standard errors of the coefficients.

Text messaging coverage is an indicator for the text messaging intervention, that takes a value between 0 and 1. If a village did not receive the text messaging intervention, its value is 0. If a village received the intervention, its value is equal to the number of phone numbers collected in that village divided by the estimated number of households in that village based on the district population size divided by the average household size. Presence of community leaders, pastoralists and livestock field officers are all binary variables, taking a value of one if the village received the intervention, if a pastoralist community resided in the village or if a livestock field Officer was permanently stationed in that village. The collective action indicator takes values between 0 and 1 and was generated from a small experiment designed to capture the capability of a village to coordinate in collective action. A higher value indicates a greater ability to coordinate in collective action. For more information, see Methodology. Text Messaging Coverage*Community Leaders is an interaction term for the two advertising interventions.

^a^Significant at 10% level.

^b^Significant at 5% level.

Next, to analyse the effect of our interventions on village-level vaccination coverage, we used general linear mixed models (GLMMs) with the logit link function and estimated vaccination coverage as our dependent variable (Table 1). In addition to the advertising interventions, we included two other explanatory variables in our first model: the presence of pastoralists and the presence of a livestock field officer stationed in a village. Pastoralists tend to own more dogs than other communities and often live more remotely, therefore may be expected to have greater difficulty accessing vaccination points (Fitzpatrick et al., 2012). We hypothesised it might make community members more trusting of animal health interventions if a livestock officer operates in their village. Only the coefficient for Community Leader advertising was statistically significant at the 5% level and positively associated with increased participation, while the effects of the presence of pastoralists and livestock field officers were as expected, negative and positive, respectively, but not statistically significant.

When we included an interaction between the two interventions (Table 1: Models 2 and 3), the coefficient for *Community Leaders* lost statistical significance. The interaction term was significant at the 5% level and a negative coefficient for *Text Messaging Coverage* was significant at the 10% level. The positive effect of the interaction between the interventions outweighed the negative effect of mobile phone text messaging, indicating that when used together, these interventions tend to be associated with increased vaccination coverage. We also explored an indicator for a community’s propensity for collective action (Table 1: Model 3, see Methods), but did not find a statistically significant effect. Other control variables were used in attempts to improve the models, but most were discarded because they had no statistical significance nor did they improve the model. Variables in Table 1 were retained because they were statistically significant or they improved the akaike information criterion (AIC). Overall, our results suggest that the interventions we tested only improved vaccination coverage when used in conjunction with each other.

We conducted in-depth interviews with 16 local villagers to further explore and understand the findings from the quantitative analyses. Five of the nine respondents who had received text-messages indicated that they needed information through other sources to confirm their authenticity. To quote one of the respondents:

“(When) the text came before the campaign, I didn’t think anything of it, but after two or three days, some people came advertising on loudspeaker about the campaign, …, realising the text was also related to this, I said to myself I must vaccinate my dogs.”

Local village/sub-village leaders and officials as well as other advertisement methods were mentioned amongst the other sources of information about the vaccination campaign, consistent with the positive interaction of the interventions (Table 1). Other factors mentioned in the interviews that may explain the lack of an effect of text messaging alone included illiteracy and weak phone signal. Despite these issues, qualitative data provided evidence of the value of text messaging. The anecdote in the text message about a recent rabies death was particularly relevant as recipients could relate the case to themselves or their family. Two respondents explicitly reported that the content of the text message made them feel differently about the risk of rabies. One said:

“… when you receive a text like this and when you look back at home, you realise you have dogs and kids, you will definitely take your dog for vaccination …”.

The other interviewee said:

“… very few people didn’t vaccinate their dogs, the majority of them were sensitised by the texts.”

These excerpts show that the content of text messaging could indeed make the risk of rabies salient to recipients.

While qualitative data provided some insight into why the text messaging intervention needed community leaders to produce a positive effect, it did not explain why the latter were ineffective on their own, nor did it explain the mechanism through which community leaders motivated community members to participate in the vaccination campaigns. Whilst respondents appreciated the use of community leaders, they did not attribute any particular influence due to the information coming from the community leaders. However, they did consider community leaders as a trusted source of information, unlike the text-messages.

### Addressing operational barriers to canine vaccination

To analyse the effect of operational barriers, we collated vaccination coverage data from 2018 with secondary coverage data from 2016 and 2014 (of the 56 villages in our experiment, we obtained data for 29 villages for 2014 and 33 villages for 2016, giving 118 total observations).

**Fig. 3:**
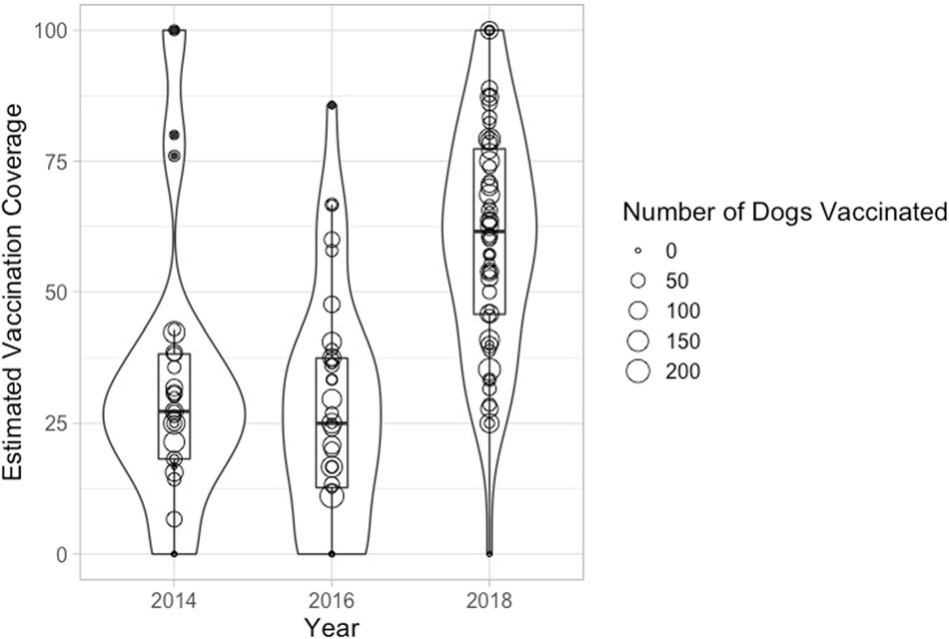
Mean and variation in vaccination coverage between years 2014, 2016 and 2018. Mean vaccination coverage was around 25% for both years 2014 and 2016, but was much higher in 2018 at approximately 60%. As far as we know, the advertising interventions and the operational changes to how the campaigns were run were the most substantive changes that could have affected participation in the vaccination campaigns. Given the results of the advertising interventions (Fig. 2), we can be confident that the improvement in coverage in 2018 can at least be partially attributed to the operational changes.

Mean vaccination coverage, measured from post-vaccination transects, was significantly higher in the 2018 campaign with the introduction of these operational changes compared to 2014 and 2016 (Fig. 3). Using regression analysis, we found that the operational changes had a highly significant and positive effect on vaccination coverage (Table 2, Model 1), but this could likewise be attributed to year-to-year variation (Table 2, Model 2). Awareness about recent rabies cases could have affected participation, but we know that human rabies deaths have occurred in the district every year, and have no reason to believe that awareness in 2018 was higher or communities were more sensitised from recent rabies cases/deaths. The only substantive changes that we know could have impacted participation were the advertising interventions and the operational changes to the campaigns. We therefore attribute the improved coverage, at least partly, to these operational changes. The effects of the advertising interventions were also the same as in the primary analysis (Table 2), except with reduced statistical significance, which was expected.

**Table 2:** Regression models analysing the effect of the operational changes on participation, with estimated vaccination coverage as the dependent variable (as per Table 1).

Independent variables	Model 1 coefficient (standard error)	Model 2
Text messaging coverage	−0.8244 (0.6136)	−0.8250 (0.6144)
Community leaders	0.1773 (0.3000)	0.1772 (0.3003)
Operational changes	1.2757^a^ (0.2403)	
Year 2016		−0.0458 (0.2362)
Year 2018		1.2516^a^ (0.2706)
Text messaging coverage*Community leaders	1.3663^b^ (0.8372)	1.3671^b^ (0.8383)

Operational Changes is a categorical variable, which takes the value one when operational changes were implemented (all observations in 2018) and zero if not (all other observations). Year is a categorical variable (2014, 2016 and 2018). In Model 1, the coefficient for Operational Changes is positive and statistically significant. In Model 2, the categorical variable for year 2018 is positive and statistically significant, but not for 2016. These results suggest that the operational changes implemented in 2018 had a positive impact, but we cannot rule out other temporal confounders in 2018.

^a^Significant at 1% level.

^b^Significant at 10.3% level.

Our qualitative data provided further insight into how operational changes improved participation. More than half of the respondents in our follow-up interviews mentioned that distance from vaccination points and difficulty restraining dogs while travelling to a vaccination point affected their participation. Shortening the distances at least partially tackled this issue. Stationing vaccination points in sub-villages also involved sub-village leaders in campaign advertising. One respondent mentioned that sub-village leaders tend to have information about dog-owners in their respective sub-village and that consequently they can also encourage participation. Over a third of respondents reasoned that having vaccination points open for a whole day gave more time and flexibility to vaccinate their dogs. Several also believed that campaigns should have been held for at least 2 days and others recommended house-to-house vaccination as their preferred strategy. However, such strategies would dramatically increase costs and none of the respondents suggested ways to address their feasibility. Extended advertising of vaccination campaigns (1 week versus 1 day) helped two thirds of the respondents prepare, by, for example, giving them time to restrain their dogs. It also allowed information dissemination within villages. Dogs in Tanzania typically freely roam. This can make it difficult for owners to restrain them at short notice. In addition, according to respondents and data on how villagers learnt about the vaccination campaigns, word-of-mouth played an important role in the transmission of information.

## Discussion and conclusion

We designed advertising interventions aiming to increase participation in canine vaccination campaigns against rabies, by informing participants about the benefits of vaccination and making the risk of rabies salient. We were interested in whether the moral authority and influence of community leaders and persuasive text-messages could have greater reach than routine advertising, and improve collective action. We found no statistical evidence that either of our advertising interventions when used alone were effective in increasing participation, but when used together we saw a positive impact. One reason for not detecting an effect of either intervention alone may have been that communities were already sufficiently informed and motivated. Indeed, we found that operational changes to vaccination campaign delivery greatly increased participation, suggesting that the risk of rabies is salient to communities and engagement can be very high, but that actors’ agency to participate is limited by practical barriers.

The lack of impact from either advertising intervention alone may have been due to contextual factors. Our results contrast those obtained in Haiti where text messaging had a significantly positive effect on dog vaccination coverage (Cleaton et al., 2018). First, in the study in Haiti almost all phone numbers in the areas of focus were obtained. It is therefore likely that a greater number of people were reached through mobile phones in Haiti compared to our study. Second, compared to the communes studied in Haiti (Gonaïves and Saint-Marc), Morogoro Rural District in Tanzania is more sparsely populated, with greater distances to vaccination points. Despite routine advertising beginning 1 week before the vaccination day, the study in Haiti reported that just 15% of interviewees heard about the campaign from others, whereas in Tanzania the corresponding figures are much higher: >63 and 50% from household and vaccination point questionnaires, respectively. The culture of communication and information sharing likely differ between these settings. Many text recipients and also non-text-recipients in Tanzania were made aware of the vaccination campaigns through other sources, whereas text messaging was the primary source of information in Haiti. Routine advertising and word-of-mouth played an important role in spreading information in Tanzania; over 88% of participants at vaccination points reported being made aware of the campaigns through routine advertising and other villagers. From household questionnaires, which were administered to 20 randomly selected dog-owning households in the 56 villages, this figure was more than 98% amongst those aware of the campaigns (11.3% of the total sample). The same contextual factors could have limited the effect of community leaders’ advertising. Other studies have shown that religious and political leaders can reduce vaccine hesitancy and increase vaccination uptake for polio and many other diseases, including rabies (Nasiru et al., 2012; Goldstein et al., 2015; Léchenne et al., 2016). Our qualitative data also showed support for community leaders as a trusted source for communication. Overall, this does not suggest that the advertising interventions were ineffective in raising awareness, but that their effects were largely redundant and context-dependent.

Our second set of results showed that operational changes to how vaccination campaigns were run could have a major impact on participation. We are cautious in interpreting these results, since we have no other information on village-level changes that could have affected participation. Nonetheless, our results are intuitive and there are many arguments to support them. The first change, stationing vaccination points at sub-village level, lowered the direct participation cost incurred by villagers. Several authors show that distance to a vaccination point impacts participation (Beyenne et al., 2018; Kaare et al., 2009). We then addressed the lack of awareness by starting to advertise vaccination campaigns well in advance. The other operational change was opening vaccination points all day long, giving villagers more flexibility and opportunities to vaccinate their dogs. Another comprehensive study showed that similar changes, that include campaign implementation and timely advertising, can significantly improve participation (Castillo-Neyra et al., 2017).

Our theoretical framework (Box 1) illustrates how the opportunity cost of participation and salience are mutually complementary factors, as they interact multiplicatively, to solve this collective action problem. The lack of support for our experimental indicator of collective action may reflect limited between-community variation in propensity for collective action, or even that villagers may not see rabies elimination as a collective action problem. However, our qualitative data suggest that communities see the individual and collective benefits from dog vaccination and the moral obligation to participate given the risks of this fatal disease. In practice, our data show that access to vaccination campaigns and costs of participation for communities can be sufficiently reduced through straightforward logistical changes enabling the majority to participate.

Our results may have broader implications beyond rabies elimination, and may apply to public good provision in general, especially those that need voluntary participation from community members, including vaccination campaigns for other infectious diseases that require a threshold coverage to achieve herd immunity (Burgess et al., 2021). We argue that although our advertising interventions could have been effective, their impacts were limited or redundant due to contextual factors. Our operational changes greatly improved participation in vaccination campaigns. These findings suggest that for cost-effective policies that deal with a collective action problem, policy-makers need to understand the local context. In our case, advertising interventions inform and make the problem salient to community members, and operational changes sufficiently reduce the costs of collective participation. One shortcoming of our research is that we were unable to isolate the effect of advertising in general, because we combined advertising interventions with other operational changes. Being able to isolate and identify each problem, either lack of information and knowledge or ineffective campaign delivery or both, may help in further designing cost-effective policies that can appropriately target contributing factors.

### Methods

Assuming each of the interventions could raise vaccination coverage at village level by 10%, we conducted a simulation-based power analysis to approximate the sample size needed to detect this effect at 80% probability. As a result, we randomly selected 56 villages from the 93 villages in the Morogoro Rural district of Tanzania that the local veterinary officers confirmed had permanent mobile phone network. We then randomly allocated the sampled villages to four groups that received different combinations of the interventions.

Approximately 2 months prior to implementing the advertising interventions, we conducted a series of workshops, aiming to identify potential issues that might impede the implementation of our interventions, and to seek local advice on improvements to the study design and vaccination campaigns more generally. Participants were recruited from 43 of the 56 villages. We did not recruit participants from the remaining 13 villages because of accessibility problems. However, district veterinary officers, who were familiar with those villages, were present during workshops in other villages and were able to provide relevant information. From each of the 43 villages, we recruited up to 3 participants: a village leader, a veterinary officer (if there was one allocated to the village) and a health official (nurse or medical attendant). A total of 15 workshops were held over a period of 8 days, with the number of participants in each workshop ranging from 4 to over 20 participants. In total, 146 participants contributed to this series of workshops. Workshop participants were first asked about their experience with past rabies vaccination campaigns, including challenges encountered. After detailing the plan related to the advertising methods to be tested, they were encouraged to provide feedback on the design and any potential impediment they envisaged. Issues encountered in previous vaccination campaigns were identified from these workshops and informed our operational changes. Participants also helped design the overall intervention, and the structure and content of text-messages and their timing for sending them.

Focus group discussions were conducted in 5 of the selected villages comprising a total of 47 villagers. Participants were mostly ordinary community members. The objective of these discussions was to produce a locally relevant message to be tested in the advertising interventions, although the overall study design was also addressed. These conversations began with a general discussion on rabies to assess participants’ experience with the disease. Feedback on the anticipated design of the study was sought. Specifically, participants were asked how to ensure that text-messages would attract attention and make the risk of rabies salient to recipients. The design of the text messaging intervention was a result of these focus group discussions and the workshops discussed earlier.

Another workshop was conducted with a group of 8 community leaders (two village leaders, one Muslim leader, three Christian leaders from different denominations (Pentecost, Tanzania Assembly of God and Lutheran) and two Maasai leaders. Leaders were selected from villages outside the study to prevent contamination if they were to take initiative to advertise vaccination campaigns without our invitation. This workshop also started with a general discussion on rabies and vaccination campaigns to familiarise the participants with the problem, followed by a discussion on the roles that leaders could play in advertising. We also clarified that the participation of local leaders in our experiment would not contradict their beliefs or traditions in any way. The design of the community leader intervention as discussed above was a result of this workshop.

In addition to these advertising interventions, routine advertising was also conducted. Posters were placed at prominent places such as schools, markets and town halls 1 week before the vaccination date and left there until at least the day after the campaign. Local village/sub-village administrative leaders also used loudspeakers for advertising. All these methods provided information on the location and timing of the vaccination campaigns, and clarified that vaccines were provided for free. Vaccinators and other officials involved were instructed on how to carry out these routine advertisements and the vaccination campaigns themselves.

We used a central point vaccination strategy (Kaare et al., 2009) in which villagers bring their dogs to a central point operated by a vaccinator. In total, there were 88 such vaccination points in the sampled villages. Larger villages with multiple sub-villages had more vaccination points according to the number of sub-villages (maximum of 3 points per village), whereas smaller villages only had one vaccination point.

Upon vaccination, dogs were marked with a coloured collar, to allow vaccinated dogs to be distinguished from unvaccinated dogs during transects conducted on the evening of each campaign. Vaccinators were trained to conduct transects in their respective villages (Sambo et al., 2017; Kaare et al., 2009). After the vaccination campaigns, the vaccinators walked through their respective villages or sub-villages and counted the number of dogs with and without collars. Vaccination coverage was estimated by dividing the number of dogs observed with collars by the total number of dogs seen during the transect.

At selected vaccination points, those who brought their dogs for vaccination were asked to complete a short survey to collect data on how they learned about the vaccination campaigns and what advertisement methods they were exposed to (Table S1).

Questionnaires were administered to village leaders (either village chairpersons or executives) in the sampled villages the following week to collect village-level socio-economic data and other variables that could have influenced coverage. The questionnaire included information on access to basic needs and facilities as well as experiences and attitudes towards rabies and previous vaccination campaigns. These interviews also allowed us to check whether the interventions were implemented as intended. As official village-level data were limited, we largely relied on village leaders’ knowledge of the variables of interest. Most of these variables were subsequently discarded from analyses because they did not improve the models in terms of AIC and provided little additional insight on vaccination campaign participation.

Household questionnaires were administered and completed within 5 weeks of the vaccination campaigns to twenty randomly selected households in each study village. In total, the questionnaires were administered to 1117 households. The questionnaires were administered to the head of each household or, if the head was not present, the most senior adult (at least 18 years old). If no adult was present, a replacement household was selected. Relevant data collected in this questionnaire included whether each household participated in vaccination campaigns, whether they knew about the campaigns and if so how they learned about the campaigns.

To create an indicator for a village’s willingness to participate in collective action, the same households were asked to engage in a small experiment. All participants were given 5000 Tanzanian Shillings (TZS) (equivalent to 2.15 USD) and were instructed to either return the cash (participate in collective action) or keep the cash (refuse to participate). If at least 14 out of 20 participants in the village returned the cash, then all participants within the same village were rewarded with 20,000 TZS (equivalent to 8.60 USD). Otherwise, there was no reward and those who returned the 5000 TZS lost the cash. The proportion of participants who returned the cash was used in regression analyses as an index for a village’s propensity to participate in collective action, such as participation in dog vaccination. It should be noted that since this was an experiment in a game theoretic setting, some kind of reward and risk was deemed necessary (Gneezy et al., 2011). However, we are aware of potential ethical concerns associated with this approach that we articulate below. The literature (Surmiak, 2020) on the ethical considerations related to giving a financial payment to research participants discusses many concerns all relevant to our research study: undue influence on voluntary consent, the commercialisation of the researcher-participant relationship, the discomfort of research participants and problems with ensuring their anonymity. We obtained ethics approval from local authorities and institutions as well as the University of Glasgow and informed consent was sought from all participants at each stage of the research including the construction of our collective action index. Nonetheless, it is appropriate to emphasise that an explicit consideration was made to take into account the needs of participants in relation to this payment specifically. We considered a number of rewards for this experiment to reduce ethics risks while enabling the assessment of willingness to participate in collective action, including provision of a limited amount of cash, mobile phone credit and food (maize). After undergoing scrutiny before local officials and researchers who had extensive experience with the living conditions in this area, cash was the most advised option and the least problematic in terms of ethical concerns and research outcomes. Issues related to mobile phone credits included skewing our sample in favour of mobile phone owners, who tend to be of higher social standing. On the other hand, food (maize) presents a safety risk to participants as we were not able to guarantee its quality and safety. Cash transfers have been widely used in Tanzania and are a familiar incentive that are considered acceptable in these communities (e.g., de Walque et al., 2012; Prencipe et al., 2021; Green, 2021; Evans et al., 2019; Kennedy et al., 2014). Therefore, for these reasons, cash rewards of the amounts specified above were agreed to be the most appropriate solution for this experiment and took into account local sensitivities based on the advice of local officials, ethics committees and researchers.

Follow-up in-depth interviews were conducted with 16 respondents selected from household questionnaires. The respondents were chosen based on the interventions they were exposed to and their decision to participate or not. Specifically, respondents who received any of the interventions were asked if the interventions affected their decision to participate or not. Respondents, who did not participate in the vaccination campaigns and were not exposed to the interventions, were asked whether the interventions could have affected their decision had they been exposed to the interventions, and whether other factors prohibited them from participating. The respondents were specifically asked if any of those operational changes made it easier for them to participate in the vaccination campaigns and if there were other changes that could have been made to improve the campaigns further.

To analyse the effect of the advertising interventions on participation, we used GLMMs with the logit link function. The use of GLMM addresses several issues. First, the dependent variable (estimated vaccination coverage) is bounded between 0 and 1. Second, the estimation of vaccination coverage using transects is likely to be noisy, especially for villages with low dog counts. The GLMM allowed us to weight regressions by dog counts and thereby use data from all 56 villages in our sample, instead of discarding villages with low dog counts. We modelled noise in the transect coverage estimates as village-level random effects, to provide more robust coefficient estimates.

To analyse the effect of the operational changes on participation, we collated the estimated vaccination coverage data of all sampled villages in 2018, 2016 and 2014. For 2016 and 2014, we had data on only 33 and 29 of the 56 villages. These provided 118 observations. The only known difference between those years and across all villages were the advertising interventions and operational changes, which we included in the analysis. The result of this analysis is at most suggestive of the effect of the operational changes on participation.

All discussions and interviews were held in the local language, Kiswahili, and facilitated by a native speaker. The conversations were recorded and subsequently transcribed and translated to English. We used thematic analysis to identify common themes mentioned in the interviews. This information was then cross-checked with our quantitative data to gain a more in-depth understanding of issues that we discovered. Data from household questionnaires were recorded using Open Data Kit on mobile Android devices.

### Supplementary information

Supplementary material

## Data Availability

Datasets collected and analysed in this paper is available in the Dataverse repository (Lim et al., 2022).
