# Supplementary material for: Tackling barriers to collective action for effective vaccination campaigns: rabies in rural Africa as an example
Source: Humanit Soc Sci Commun. 2022 Oct 12;9(1):364. doi: 10.1057/s41599-022-01374-3 (PMC11076219; doi:10.1057/s41599-022-01374-3)
Supplement: Supplementary file 1 — Supplementary material [file 41599_2022_1374_MOESM1_ESM.docx]

**Supplementary Information**

Table S1. Respondents exposed to different advertising methods based on vaccination surveys

| Number *(percentage)* of respondents exposed to different advertising methods by experimental group | Experimental Groups | | | | **Total** |
| --- | --- | --- | --- | --- | --- |
|  | Routine advertisement only | Routine advertising plus community leaders only | Routine advertising plus text messaging only | Routine advertising plus both interventions |  |
| Awareness methods: |  |  |  |  |  |
| Text messaging only | N/A | N/A | 66 | 21 | 87 *(9.81%)* |
| Community leaders only | N/A | 12 | N/A | 1 | 13 *(1.47%)* |
| Routine advertising only | 117 | 59 | 54 | 39 | 269 *(30.32%)* |
| Other villagers only | 81 | 42 | 79 | 50 | 252 *(28.41%)* |
| Text messaging plus community leaders only | N/A | N/A | N/A | 3 | 3 *(0.34%)* |
| Text messaging plus routine advertising only | N/A | N/A | 19 | 15 | 34 *(3.83%)* |
| Text messaging plus other villagers only | N/A | N/A | 16 | 13 | 29 *(3.27%)* |
| Community leaders plus routine advertising only | N/A | 24 | N/A | 4 | 28 *(3.16%)* |
| Community leaders plus other villagers only | N/A | 7 | N/A | 5 | 12 *(1.35%)* |
| Routine advertising plus other villagers only | 52 | 13 | 28 | 7 | 100 *(11.27%)* |
| Text messaging plus routine advertising and other villagers only | N/A | N/A | 16 | 9 | 25 *(2.82%)* |
| Community leaders plus routine advertising and other villagers only | N/A | 11 | N/A | 12 | 23 *(2.59%)* |
| Both advertising interventions plus routine advertising only | N/A | N/A | N/A | 7 | 7 *(0.79%)* |
| Both advertising interventions plus other villagers only | N/A | N/A | N/A | 2 | 2 *(0.23%)* |
| All methods only | N/A | N/A | N/A | 3 | 3 *(0.34%)* |
| **Total** | 250 | 168 | 278 | 191 | 887 |

Table S2. Respondents exposed to different advertising methods based on household questionnaires

| Number *(percentage)* of respondents by experimental group and awareness methods | Experimental Groups | | | | **Total** |
| --- | --- | --- | --- | --- | --- |
|  | Routine advertisement only | Routine advertising plus community leaders only | Routine advertising plus text messaging only | Routine advertising plus both interventions |  |
| Awareness methods: |  |  |  |  |  |
| Text messaging only | N/A | N/A | 11 | 2 | 13 *(1.12%)* |
| Community leaders only | N/A | 1 | N/A | 2 | 3 *(0.27%)* |
| Routine advertising only | 68 | 52 | 42 | 52 | 214 *(19.21%)* |
| Other villagers only | 131 | 127 | 119 | 118 | 495 *(44.43%)* |
| Text messaging plus community leaders only | N/A | N/A | N/A | 1 | 1 *(0.09%)* |
| Text messaging plus routine advertising only | N/A | N/A | 21 | 18 | 39 *(3.50%)* |
| Text messaging plus other villagers only | N/A | N/A | 41 | 16 | 57 *(5.12%)* |
| Community leaders plus routine advertising only | N/A | 4 | N/A | 0 | 4 *(0.36%)* |
| Community leaders plus other villagers only | N/A | 2 | N/A | 2 | 4 *(0.36%)* |
| Routine advertising plus other villagers only | 35 | 41 | 27 | 31 | 134 *(12.03%)* |
| Text messaging plus routine advertising and other villagers only | N/A | N/A | 2 | 7 | 9 *(0.81%)* |
| Community leaders plus routine advertising and other villagers only | N/A | 5 | N/A | 1 | 6 *(0.54%)* |
| Both advertising interventions plus routine advertising only | N/A | N/A | N/A | 0 | 0 *(0%)* |
| Both advertising interventions plus other villagers only | N/A | N/A | N/A | 0 | 0 *(0%)* |
| All methods only | N/A | N/A | N/A | 1 | 1 *(0.09%)* |
| Not aware of the campaigns or not exposed to any advertising | 46 | 44 | 15 | 29 | 134 *(12.03%)* |
| **Total** | 280 | 276 | 278 | 280 | 1,114 |
